# Treatment of Minimal Residual Disease in Breast Cancer: A Longitudinal Case Study

**DOI:** 10.7759/cureus.1521

**Published:** 2017-07-27

**Authors:** Nigel P Murray

**Affiliations:** 1 CTC Unit, Universidad Finis Terrae

**Keywords:** breast cancer, minimal residual disease, circulating tumor cells, treatment, micrometastases

## Abstract

The presence of micrometastatic disease will ultimately determine the breast cancer-specific mortality of patients treated according to current guidelines. Minimal residual disease (i.e., occult tumor, not detected by conventional tests) may exist in two forms: a dormant form of only micrometastasis and a more aggressive “awakened” form where CTCs (circulating tumor cells) are actively disseminating. The hypothesis is that patients with CTCs have a more advanced or aggressive disease (that the cancer has “awoken” and there is active dissemination), whereas those patients with only micrometastasis have “dormant” disease and, although at risk of future relapse, may not do so for many years. This case study shows how determining the presence of both CTCs and bone marrow micrometastasis could be used to monitor disease activity and determine treatment changes before the appearance of metastatic disease.

Presented is the case of a 53-year-old postmenopausal woman who presented with a T2N1M0 invasive ductal breast cancer. She had been treated with partial mastectomy, axillary dissection, local radiotherapy, and adjuvant chemotherapy. As the cancer was estrogen receptor-positive, she was taking tamoxifen. Two years into treatment, she was assessed for minimal residual disease and was found to be positive for CTCs and bone marrow micrometastasis. Her treatment was changed to letrozole and differing bisphosphonates. The minimal residual disease was finally eliminated, and at 16 years post-initial treatment, there was no evidence of relapse.

The detection of minimal residual disease can be used to monitor treatment effect and change therapy in order to maintain the asymptomatic status of the patient and prevent disease progression.

## Introduction

The presence of micrometastatic disease will ultimately determine the breast cancer-specific mortality of patients treated according to current guidelines. Early in the disease process, tumor cells disseminate into the circulation, with an estimated 10^6 ^circulating tumor cells (CTCs) per gram of primary tumor entering the circulation on a daily basis. However, less than 0.01% of these CTCs will survive, being destroyed by shear forces within the circulation or not having the phenotypic characteristics to implant and survive in distant tissues [1]. Breast cancer CTCs show osteotropism, targeting the hematopoietic stem cell niche in the bone marrow where they implant. Once implanted, there is an interplay between the tumor cells and stromal microenvironment. The tumor cells may remain dormant for prolonged periods of time. Factors underlying this phenomenon include balanced proliferation and apoptosis, angiogenic suppression, and immunosurveillance [2]. Dormant tumor cells retain the capacity to proliferate, but by definition, they are not currently dividing. Hence, they are resistant to treatments targeting cell division. Dormancy is seen in the clinical practice as the prolonged clinical disease-free survival between removal of the primary tumor and disease recurrence, which is frequent in breast cancer. This process is dynamic in nature; changes in the tumor cells and/or microenvironment lead to an “awakening” of dormant tumor cells, which can reenter the circulation where they are detected as secondary CTCs. Thus, this minimal residual disease, i.e., occult tumor not detected by conventional tests, may exist in two forms: a dormant form of only micrometastasis and a more aggressive “awakened” form where CTCs are actively disseminating.

The hypothesis is that patients with CTCs have a more advanced or aggressive disease where the cancer has “awoken*”* and treatment changes should be considered. Those patients with only micrometastasis have “dormant” disease and, although at risk of future relapse, may not do so for many years. Thus, those patients may be considered for treatment changes. Patients negative for minimal residual disease (within the limits of the test) for both CTCs and micrometastasis would have the best prognosis and consequently would not warrant treatment changes.

According to current guidelines, this case study shows that determining the presence of both CTCs and bone marrow micrometastasis in a patient treated for breast cancer could be used to monitor disease activity and determine treatment changes before the appearance of metastatic disease.

## Case presentation

In January 2000, a 53-year-old post-menopausal woman underwent core biopsy of the left breast for microcalcifications detected on routine mammography. The results showed an invasive ductal carcinoma; immunohistochemistry showed estrogen receptor positivity in 85%, progesterone receptor positivity in 70%, HercepTest™ (Dako, Denmark) negative, and Ki-67 positivity in 20%. According to the 2000 guidelines, she underwent partial mastectomy and axillary dissection. The surgical specimen showed 1/18 lymph nodes positive for cancer, without penetration of the lymph node capsule (pathological stage T2N1M0). She underwent locoregional radiotherapy and received four cycles of anthracycline-cyclophosphamide-based chemotherapy. After completing treatment, she was started on tamoxifen, 20 mg/day, with a planned treatment duration of five years. In the posterior controls, there was no evidence of metastatic disease or local recurrence.

In December 2002, the patient presented at our center. There was no evidence of disease relapse using CT imaging, bone scan, and mammography. For the detection of minimal residual disease, blood samples for circulating tumor cells and bone marrow biopsy for micrometastasis were taken:

a) Detection of secondary circulating tumor cells: 8 mL of venous blood was collected in an EDTA (ethylenediaminetetraacetic acid) BD-Vacutainer® (Becton, Dickinson & Co., Franklin Lakes, NJ) and the mononuclear cells were obtained using differential gel centrifugation (Histopaque®-1077; Sigma-Aldrich Corp., St. Louis, MO). The cells were washed in phosphate buffered saline pH 7.4 (PBS), then re-suspended in 100 𝜇L of autologous plasma. Twenty-five 𝜇L aliquots were used to make silanized slides (Dako, Denmark), which were dried in air for 24 hours and then fixed in a solution of 70% ethanol, 5% formaldehyde, and 25% PBS for five minutes and finally washed three times using PBS.

Immunochemistry: Secondary CTCs were detected using a monoclonal antibody directed against mammaglobin (Novocastra Laboratory, Newcastle, UK) and identified using an alkaline phosphatase - anti-alkaline phosphatase-based system (LSAB2) (Dako, Denmark) with new fuchsin as the chromogen. Positive samples underwent a second process with anti-CD45 clone 2B11 and PD7/26 (Dako, Denmark) and were identified with a peroxidase-based system (LSAB2; Dako, Denmark) with 3,3′-diaminobenzidine-tetrahydrochloride-dihydrate (DAB) as the chromogen. A secondary CTC was defined as a cell that expressed mammaglobin but not CD45, according to the criteria of the International Society of Hematotherapy and Genetic Engineering (ISHAGE) [3]. A leukocyte did not express mammaglobin but expressed CD45. A test was considered positive for secondary CTCs when at least 1 cell/8mL of blood sample was detected; the number of CTCs detected/8 ml blood sample was registered.

b) Detection of bone marrow micrometastasis: A bone marrow biopsy was taken from the posterior superior iliac crest and the sample used to prepare four ”touch preps” using silanized slides (Dako, Denmark). The slides were processed in the same way as for CTCs.

**Figure 1:**
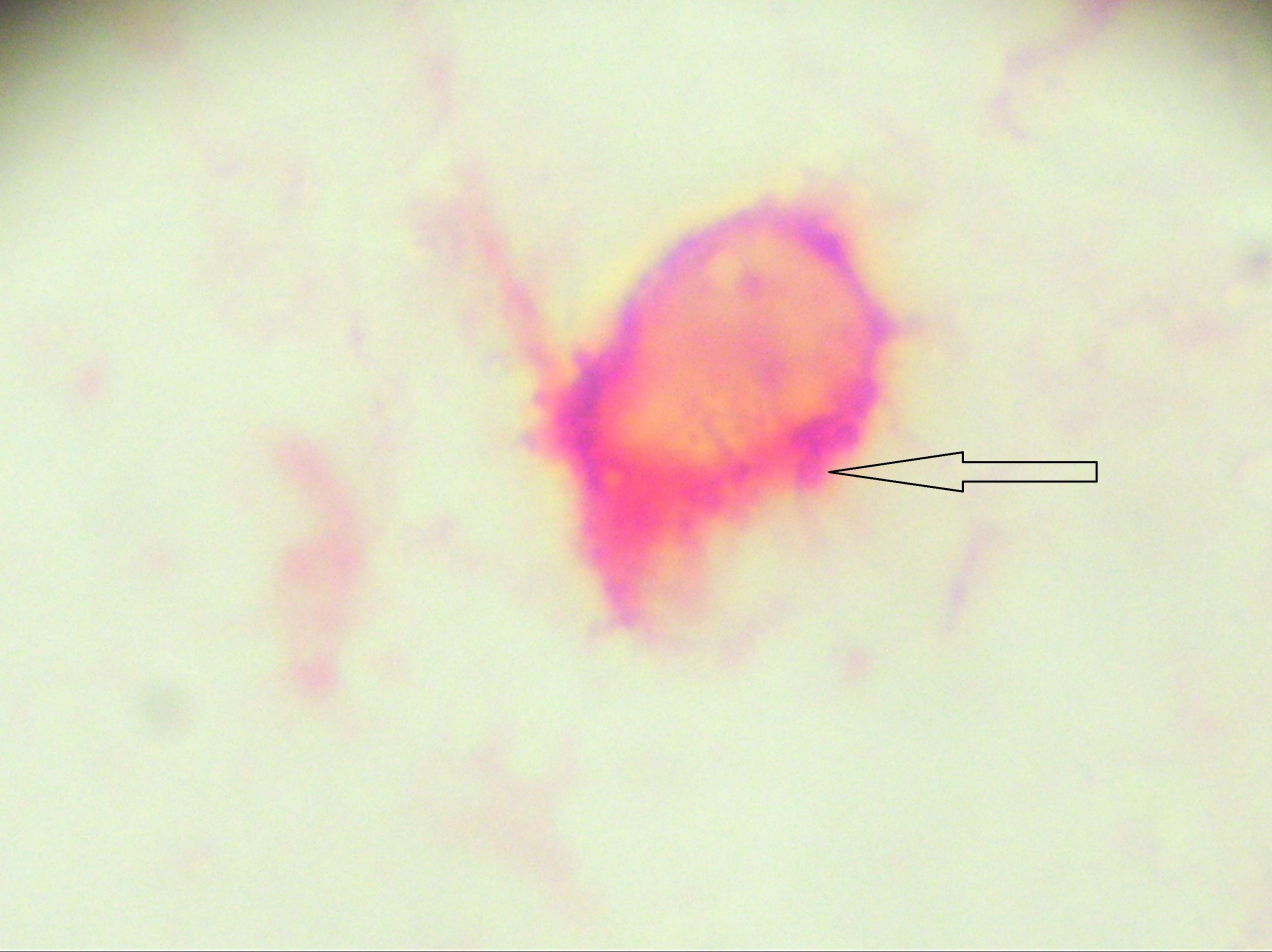
Circulating tumor cell staining positive (red) for mammaglobin and negative (black) for membrane CD45

**Figure 2:**
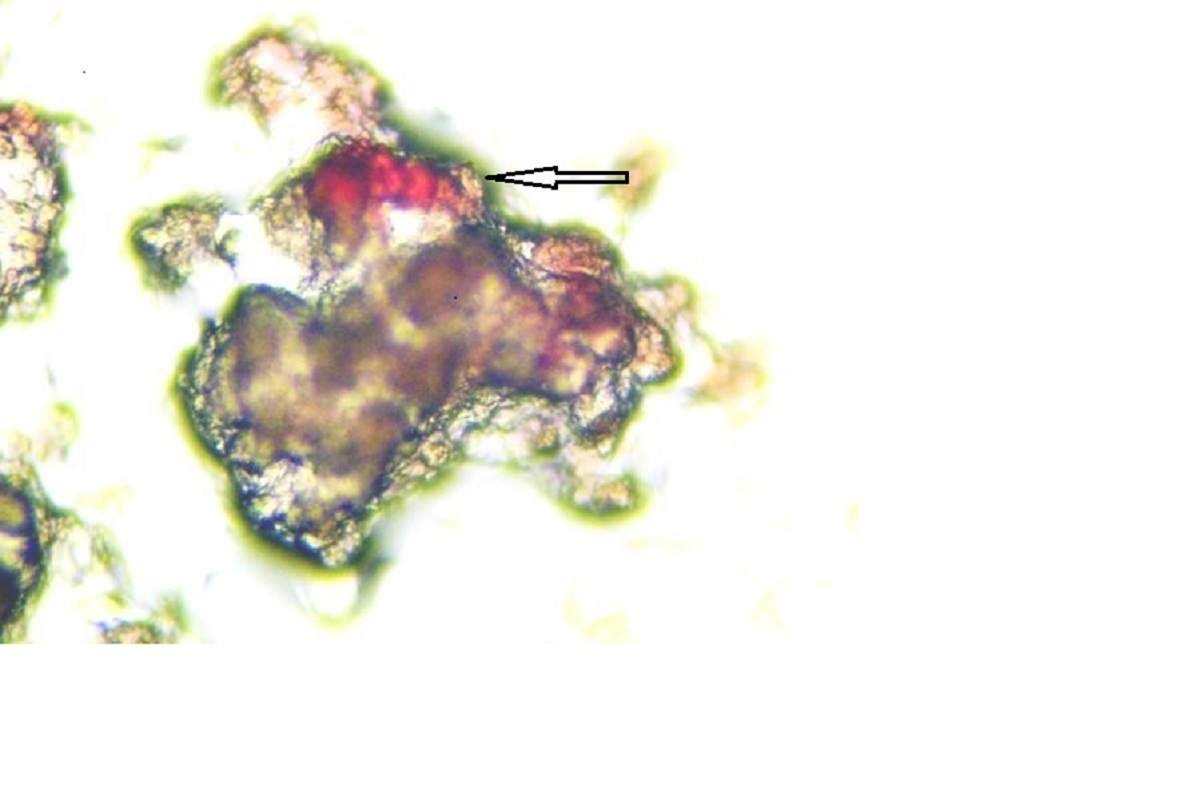
Bone marrow micrometastasis, breast cells staining red for mammaglobin (arrow) and normal bone marrow cells negative

The initial evaluation for minimal residual disease showed that the patient was positive for CTCs, with 5 cells/blood sample detected (Figure 1) and positive for bone marrow micrometastasis (Figure 2).

The results were interpreted as minimal residual disease positive with a high risk of disease progression. Her treatment was changed from tamoxifen to the aromatase inhibitor, letrozole, at a dose of 2.5 mg/day. The oral bisphosphonate, alendronate, was added at a dose of 70 mg once weekly.

**Figure 3:**
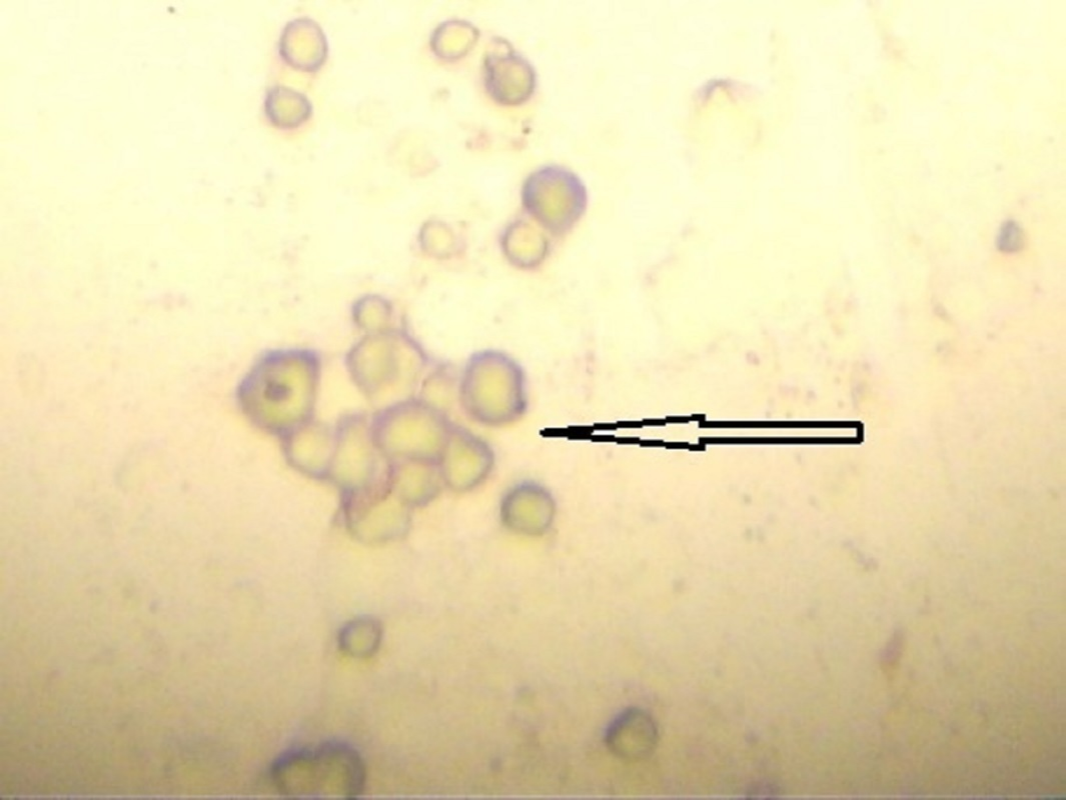
Leukocytes staining negative for mammaglobin (red) and positive (black) for membrane CD45

After six months of treatment, the patient was reevaluated. There were no CTCs detected (Figure 3).

**Figure 4:**
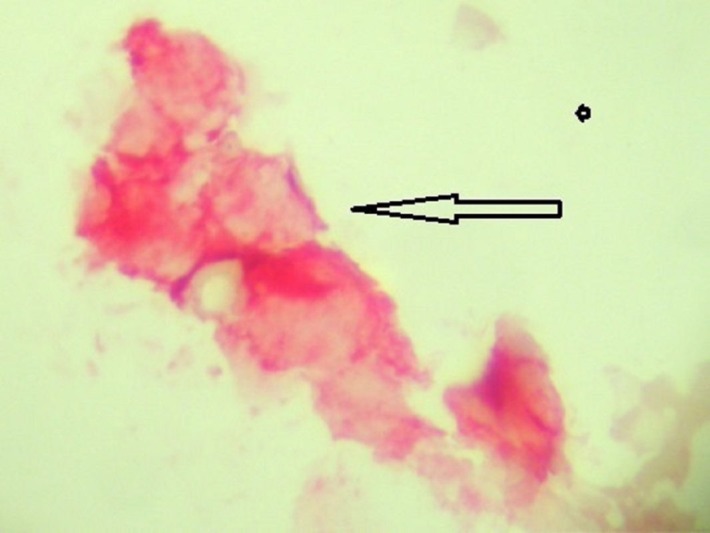
Bone marrow micrometastasis; breast cells staining red for mammaglobin and not staining for membrane CD45 (black)

The number of micrometastatic cells had significantly decreased, but the bone marrow was still positive (Figure 4).

**Figure 5:**
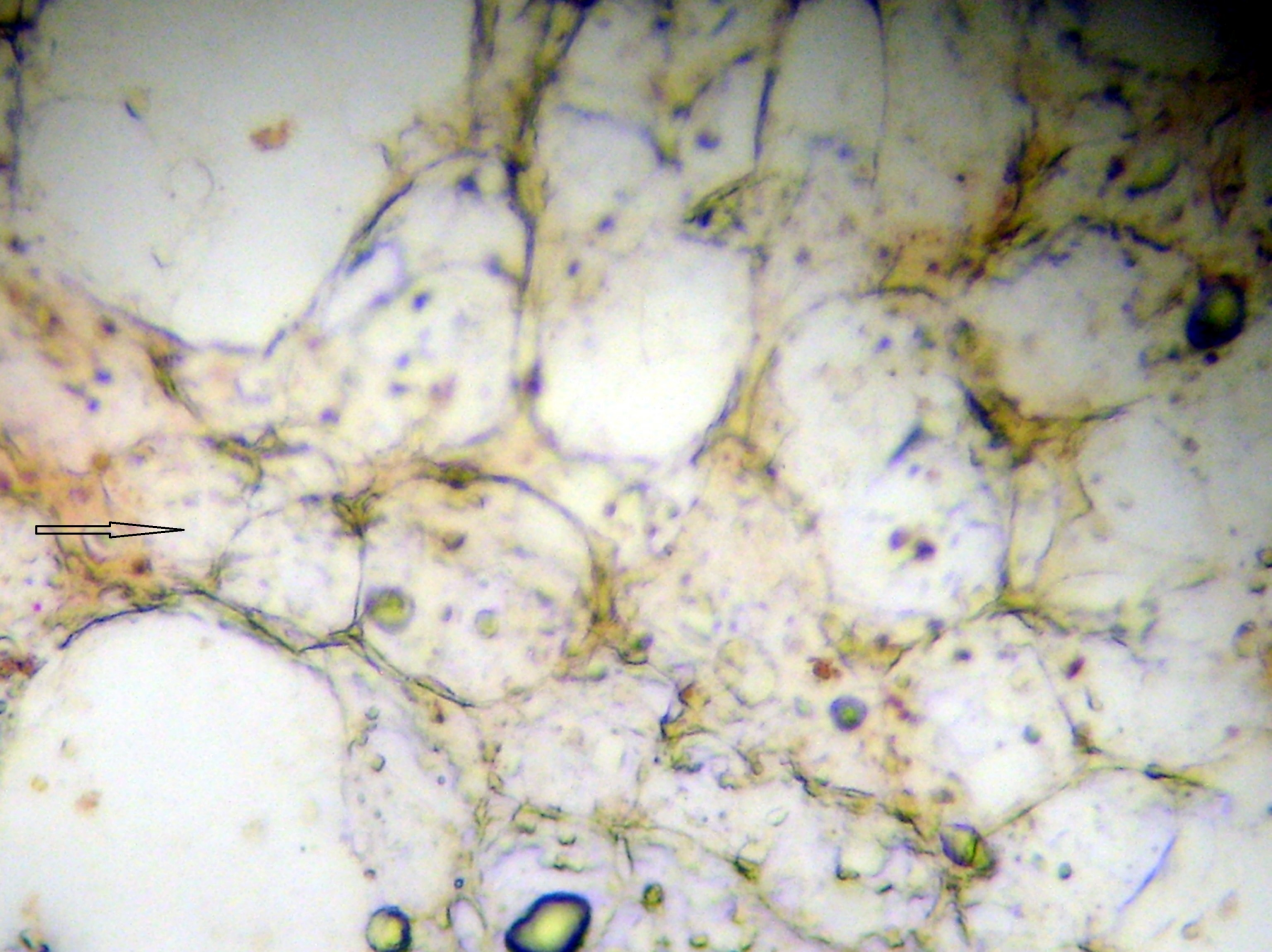
Bone marrow negative for mammaglobin staining (red) breast cells

As such, the dose of alendronate was increased to 70 mg twice weekly and the letrozole continued at the same dose. One year later, there was no evidence of CTCs or bone marrow micrometastasis (Figure 5), and treatment was continued at the same dose.

The follow-up evaluation in 2005 showed limited numbers of mammaglobin-positive cells in the bone marrow, but the patient remained negative for CTCs. There was no evidence of metastatic relapse on bone scan, CT scanning, or mammography. The alendronate was suspended and replaced for eight monthly cycles of intravenous zoledronic acid, 4 mg. After eight cycles of zoledronic acid, there was no evidence CTCs or bone marrow micrometastasis. The patient continued treatment with letrozole, 2.5 mg/day, until completing the recommended five years of treatment. The patient has remained negative both for CTCs and bone marrow micrometastasis to date; her last control was in 2016 with no evidence of minimal residual disease or evidence of metastases on imagining studies. The changes in treatment appeared to eradicate both circulating tumor cells and bone marrow micrometastasis.

## Discussion

The use of systemic adjuvant therapy has improved the survival of patients with early breast cancer. In 2002, there was a lack of established methods to identify patients with minimal residual disease or to monitor the effects of treatment in these patients. The identification of tumor cells and their characteristics opens the possibility of monitoring treatment responses and changing treatment before the development of metastasis.

In 2002, there were reports that the presence of bone marrow micrometastasis was associated with decreased survival in breast cancer patients [4]. Furthermore, there was a lack of effect of adjuvant chemotherapy on the elimination of these cancer cells [5]. Dormancy protects cancer cells from the effects of chemotherapy; the low expression of proliferation markers, such as Ki-67 and p120, in these cells implies that cell-cycle independent treatment modalities may be more appropriate in an effort to eliminate them.

We hypothesized that patients with CTCs had a more aggressive disease that had escaped from its dormant state. The cancer cells were able to reenter the circulation from the micrometastasis, implant in distant sites, and form new metastases. In this clinical case, the patient had completed surgery, local radiotherapy, and chemotherapy. She had been treated with tamoxifen for two years, had no evidence of metastasis using standard imaging, and was asymptomatic. The evaluation for minimal residual disease showed that the patient was positive for bone marrow micrometastasis and CTCs with the inference that the patient was at high risk for the development of metastatic disease. Tamoxifen and the previous systemic therapy had failed to eliminate these cells, and as such, a change in treatment was indicated. 

In 2002, the National Cancer Institute of Canada Clinical Trials Group MA.17 trial comparing letrozole versus placebo after five years of tamoxifen for estrogen receptor-positive breast cancer was recruiting patients. Letrozole had been approved for the treatment of post-menopausal women with hormone receptor-positive breast cancer that was progressing after antiestrogen (tamoxifen) therapy and as a first line treatment in 2001. In this case, the use of letrozole would be indicated as, at a microscopic level, there was evidence of progressive disease or at least high risk of progressive disease in a cancer that was estrogen receptor-positive. The reason for adding a bisphosphonate was two-fold; firstly, to prevent osteoporosis, there was reported concern that letrozole increased the risk of osteoporosis, and as such, alendronate, 70 mg once weekly, was added in the standard dose to prevent osteoporosis. More importantly, there was evidence that bisphosphonates exerted an anti-cancer effect. Studies using clodronate had reported that there were fewer bone metastases during the treatment period, although visceral metastasis was not affected, and that there was an improvement in overall survival [6]. Nitrogen-containing bisphosphonates, such as alendronate, affect the mevalonate pathway by inhibiting the post-translational prenylation of GTP-binding proteins and, as a result, cause apoptosis. They also inhibit the activation of pro-matrix metalloproteinase 1, which decreases the activation of matrix metalloproteinase 2, important in tumor dissemination. This effect of bisphosphonates is dose-dependent.

After six months of treatment, CTCs were no longer detected and the micrometastatic tumor burden had been greatly reduced in the bone marrow. Imaging studies did not demonstrate the presence of macrometastases. The inference was that, within the limits of the detection methods, the disease had regressed to the “dormant” stage and the tumor burden had been reduced. As the therapeutic effect of bisphosphonates is dose-dependent, the therapy with alendronate was increased to 70 mg twice weekly with monitoring of renal function and serum calcium levels. The treatment was well tolerated and the patient remained asymptomatic.

The control at 18 months showed no evidence of minimal residual disease with normal renal function and serum calcium levels. There was no evidence of metastatic disease on imaging. The implication was that treatment changes had eradicated both circulating tumor cells and bone marrow micrometastasis (one possible source of CTCs).

By 2005, the utility of bone marrow micrometastases as a prognostic factor had been reported, including a pooled analysis of over 3,000 patients [7]. However, there was no consensus on what treatment should be offered to these patients or, if the CTCs or micrometastasis were eliminated, whether progression-free survival and overall survival would be improved. Eliminating CTCs, as is seen in this case, does not signify that micrometastatic disease is also eliminated. The two tests are complementary in that the clinical implications of CTCs and bone marrow micrometastasis are different. At the same time, the first reports from the MA.17 trial showed that letrozole after tamoxifen improved disease-free, distant disease-free, and overall survival in node-positive women when compared with placebo [8].

Evaluation of the patient's blood and bone marrow at the end of 2005 showed the absence of detectable CTCs but mammaglobin-positive cells were again detected in the bone marrow. There was no evidence of macrometastatic disease on imaging and the patient was asymptomatic. Alendronate is an oral bisphosphonate with a variable absorption, and as such, in the dose that the patient was receiving, the pharmacological effect may not have been optimum. Consequently, the treatment was changed to intravenous bisphosphonate zoledronate monthly for eight cycles - the same treatment schedule as in metastatic breast cancer. Reevaluation of the bone marrow after eight cycles did not detect bone marrow micrometastasis, and further treatment with zoledronate was not considered. The letrozole was continued for a total of five years as recommended.

The patient was last reviewed in 2016, 14 years after starting treatment. She was currently asymptomatic and without treatment with no evidence of minimal residual disease; imaging studies did not detect metastasis.

During the follow-up of this patient, there have been numerous publications on the use of CTCs in metastatic and non-metastatic breast cancer patients, two of which are of interest in the management of this patient. Firstly, a study of women with Stage IV breast cancer where both bone marrow micrometastasis and CTCs were detected using similar methods. This study concluded that the prognosis of these women was dependent on the presence of CTCs rather than bone marrow micrometastasis [9]. Indirectly, this study supports our initial hypothesis that immunocytochemical staining of bone marrow micrometastasis does not differentiate between dormant and “awakened” tumor cells. However, the combination with CTCs identifies a group of patients with more aggressive minimal residual disease. Secondly, prospective clinical trials have reported a positive effect of zoledronate on micrometastasis and improved survival rates [10].

## Conclusions

In summary, this clinical case highlights the use of personalized medicine and that by using sequential treatment with low toxicity and a mechanism of action that is cell-cycle independent, minimal residual disease can be eliminated. It would be more correct to say that minimal residual disease was not detected, taking into consideration the limits of the detection method. However, it may not be necessary to eliminate every last tumor cell; if the cancer remains in a dormant state, the disease is converted into an asymptomatic chronic phase. This may be acceptable to the patient; if the disease “awakens” at a future time, it may be treated while in a microscopic phase before the appearance of metastasis in imaging studies. In this clinical case, the changes in treatment eliminated both CTCs and bone marrow micrometastasis. Due to treatment changes during follow-up, it would be difficult to design a prospective randomized trial; however, a real world observational study could provide answers to the questions raised.

The use of the combination of CTCs and micrometastasis can be implemented in a routine immunocytochemistry laboratory of a district general hospital and does not require high-cost technology. It provides clinically useful information to guide treatment decisions before the appearance of metastatic disease. This provides a “personalized medicine” protocol of treatment which can be modified during follow-up.
